# Preoperative and Postoperative Bone Mineral Density Change and Risk Factor Analysis in Patients with a GH-Secreting Pituitary Adenoma

**DOI:** 10.1155/2019/2102616

**Published:** 2019-11-03

**Authors:** Li'nan Qin, Xiaopeng Guo, Lu Gao, Zihao Wang, Chenzhe Feng, Kan Deng, Wei Lian, Bing Xing

**Affiliations:** ^1^Department of Neurosurgery, Peking Union Medical College Hospital, Chinese Academy of Medical Sciences and Peking Union Medical College, Beijing 100730, China; ^2^China Pituitary Disease Registry Center, Chinese Pituitary Adenoma Cooperative Group, Beijing 100730, China; ^3^Key Laboratory of Endocrinology of National Health and Family Planning Commission, Beijing 100730, China

## Abstract

**Purpose:**

This study analysed changes in bone mineral density (BMD) at different sites in patients with acromegaly and postoperative BMD changes and explored risk factors associated with BMD.

**Methods:**

Clinical data of 39 patients with growth hormone- (GH-) secreting pituitary adenomas and 29 patients with nonfunctioning pituitary adenomas who were newly diagnosed in neurosurgery from January 2016 to December 2018 were retrospectively analysed, including measurements of preoperative and postoperative BMD, serum GH glucose inhibition, random GH and IGF-1, and other anterior pituitary hormones.

**Results:**

The average patient age and disease duration were 43.74 (33.41–54.07) years and 72.15 (22.82–121.48) months, respectively. Compared with patients with nonfunctioning adenomas, patients with GH-secreting pituitary adenomas had significantly higher BMDs at L1, L2, femoral neck, Ward triangle, trochanter, femoral shaft, and total hip sites (*p* < 0.05). The BMD *Z* score at L1 and femoral neck sites significantly increased (*p* < 0.05). Thirteen patients underwent re-examination of BMD 1 year postsurgery, and the BMD *Z* score was reduced to normal levels at L1, L2, L3, L4, L1-L4, and L2-L4 compared with preoperative levels (*p* < 0.05). Postoperative BMD *Z* scores in the femoral neck and total hip were significantly increased (*p* < 0.05). Disease duration was negatively correlated with the lumbar-spine BMD *Z* score. IGF-1 burden was negatively correlated with the BMD *Z* score at L1 and L1–L4. Multiple regression analysis showed that IGF-1 burden was a risk factor for a BMD *Z* score decrease at L1 and L1–L4.

**Conclusion:**

BMD in patients with GH-secreting pituitary adenomas (compared with nonfunctional adenomas) increased at L1, L2, femoral neck, Ward triangle, trochanter, femoral shaft, and total hip sites. Lumbar-spine BMD *Z* score recovered to normal levels postsurgically when GH and IGF-1 levels were controlled. BMD *Z* score was negatively correlated with disease duration and IGF-1 burden in patients with GH-secreting pituitary adenomas, and IGF-1 burden was an independent risk factor for reduced lumbar-spine BMD *Z* score.

## 1. Introduction

Acromegaly is an endocrine and metabolic disorder syndrome caused by the oversecretion of growth hormone (GH) and insulin-like growth factor-1 (IGF-1), which results in excessive growth of bones, soft tissues, and internal organs. The structure and function of bones in patients with acromegaly are affected [1]. Among patients with acromegaly, 58% have osteoporosis of the lumbar spine, 74% have osteoporosis of the femoral neck, and the incidence of vertebral fracture is approximately 39% [2]. More than 95% of acromegaly cases are caused by a GH-secreting pituitary adenoma [3]. GH and IGF-1 have anabolic effects on bone metabolism that can lead to increased bone formation and resorption [4]. However, the changes in bone mineral density (BMD) in patients with acromegaly have been controversial in the past few decades. Most studies have shown that patients have increased BMD, and some studies have revealed no significant changes; however, a few studies have reported decreased bone mineral density [5–8]. We suppose that, after surgery, the BMD should change, as the elevated GH and IGF-1 levels are controlled. To date, there is a lack of conclusive evidence that the BMD in patients with acromegaly decreases after surgery. Therefore, we aimed to investigate the preoperative BMD and to determine the postoperative BMD changes in patients with acromegaly in the Chinese population.

In addition, GH mediates bone metabolism through IGF-1, which is produced in the liver [9]. The IGF-1 level is considered positively correlated with BMD in patients with acromegaly [10–12]. The effect of IGF-1 on cortical bone seems to be more dominant than that on trabecular bone in mouse models [13]. Therefore, we aimed to examine the correlation between IGF-1 levels and BMD in humans and to explore other factors that affect BMD in patients with acromegaly.

The purpose of this study was to investigate the preoperative BMD, to analyse the effect of acromegaly cure on BMD through successful surgery, and to explore risk factors associated with BMD in patients with acromegaly.

## 2. Materials and Methods

### 2.1. Study Population

Patients first diagnosed with a GH-secreting pituitary adenoma or nonfunctioning pituitary adenoma at Peking Union Medical College Hospital (PUMCH) from January 2016 to December 2018 were studied. The inclusion criteria were as follows: (1) endocrine diagnostic criteria: elevated serum IGF-1 level and lack of suppression of GH to <1 *μ*g/L following documented hyperglycaemia during an oral glucose load [3]; (2) pituitary enhanced MRI confirming a space-occupying lesion in the sellar region; (3) typical clinical manifestations of acromegaly; (4) pathologically confirmed GH-secreting adenoma; (5) performance of a BMD examination through DXA; and (6) no history of medical treatment or radiation. Patients with a nonfunctioning pituitary adenoma were selected as the control group. The inclusion criteria of control group were as follows: (1) endocrine diagnostic criteria: without hormone oversecretion for each specific pituitary tumour type [14]; (2) clinically diagnosed with a nonfunctioning adenoma; and (3) performance of a BMD examination through DXA before surgery. The exclusion criteria were (1) diagnosis of neoplastic disease; (2) diagnosis of osteoporosis-related disease, such as PCOS, hyperthyroidism, and hyperparathyroidism; (3) use of drugs that affect BMD or bone metabolism; or (4) chronic liver or kidney diseases. Disease control was defined as random GH <1 *μ*g/L or nadir GH after OGTT <0.4 *μ*g/L and age-sex normalized IGF-1 [15].

Informed consent was obtained from each patient prior to enrolment. This study was approved by the Ethics Committee of PUMCH at the Chinese Academy of Medical Sciences and Peking Union Medical College.

### 2.2. Biochemical Measurement

The methods for hormone evaluation are described in our previously published article [16].

### 2.3. BMD

BMD was measured at the lumbar spine (L1–L4), femoral neck, Ward triangle, trochanter, femoral shaft, and total hip by dual-energy X-ray absorptiometry (Lunar Prodigy Advance, Lunar Corporation, Madison, WI, USA). The T-score is defined as the difference between the measured BMD and the bone peak in young people of the same sex. The *Z* score is defined as the difference between the measured BMD and the average BMD of people of the same age and race. The BMD examination was performed after hospital admission but before the surgery or at least 3 months after surgery during clinical re-examination.

### 2.4. Statistical Analysis

All data are expressed as the mean ± SD. Student's *T* and Mann–Whitney's *U* tests were used to compare the GH-secreting pituitary adenoma group and the nonfunctional group depending on whether it was normally distributed. A paired *T* test was used to compare the preoperative and postoperative group BMD values. Pearson's correlation coefficient or Spearman's rank order was assessed to determine the correlations between BMD and other parameters. Stepwise multiple linear regression was conducted to identify potential predictive factors for BMD at each site. Statistical significance was accepted with a *p* value <0.05.

## 3. Results

### 3.1. Patient Characteristics

Thirty-nine patients with GH-secreting pituitary adenomas and 29 patients with nonfunctioning pituitary adenomas were included. Thirteen patients with GH-secreting pituitary adenomas had controlled disease and underwent re-examination of BMD 1 year after surgery. Characteristics of the study population are shown in Table 1. The age and body mass index (BMI) of the two groups were matched. The average disease duration of patients with GH-secreting pituitary adenomas was 72.15 (22.82–121.48) months, and the average adenoma size was 15.97 (9.1–22.84) mm. GH and IGF-1 levels in patients with GH-secreting pituitary adenomas were significantly higher than those in the control group (both *p* values <0.001). In addition, FT3 (free triiodothyronine), FT4 (free thyroxine), and phosphorus levels were increased significantly (*p* < 0.05, respectively).

### 3.2. BMD

#### 3.2.1. BMD in the GH-Secreting Pituitary Adenoma Group and Nonfunctioning Adenoma Group

The BMD comparison between the GH-secreting pituitary adenoma group and controls is shown in Table 2. In the GH-secreting pituitary adenoma group, BMD was significantly elevated at the L1, L2, femoral neck, Ward triangle, trochanter, femoral shaft, and total hip sites compared with the corresponding values in patients with nonfunctioning adenomas (*p*=0.006, 0.032, 0.001, 0.036, 0.005, 0.037, and 0.008, respectively). There were no significant differences at other sites.

In terms of the *Z* score, the BMD at L1 and the femoral neck were significantly elevated in patients with a GH-secreting pituitary adenoma compared with controls (*p*=0.048 and 0.022, respectively). There were no significant differences at other sites.

#### 3.2.2. Preoperative and Postoperative BMD in Patients with GH-Secreting Pituitary Adenomas

Thirteen patients with GH-secreting pituitary adenomas underwent re-examination of BMD 1 year after surgery, and the comparison of the preoperative and postoperative BMD is shown in Table 3. Compared with that of the preoperative BMD, the *Z* score of the postoperative BMD decreased significantly at L1, L2, L3, L4, L1–L4, and L2–L4 (*p*=0.037, 0.006, 0.029, 0.031, 0.012, and 0.012, respectively). The *Z* score of postoperative BMD was significantly elevated in the femoral neck and total hip (*p*=0.019 and 0.040, respectively). There were no significant differences at other sites.

#### 3.2.3. Analysis of the Potential Determinants of BMD

Pearson's correlation analysis was performed, and the results are shown in Table 4. A negative correlation was found between the disease duration and *Z* score of the lumbar spine, including L1, L2, L3, and L4 sites (all *p* < 0.05). In addition, the disease duration was also negatively correlated with the *Z* score in the trochanter and total hip (*p*=0.048 and 0.044, respectively). IGF-1 burden was negatively correlated with the *Z* score at L1 and L1–L4 (*p*=0.042 and 0.048, respectively). The PRL level was negatively correlated with the *Z* score of the femoral neck and trochanter (all *p* < 0.05). GH nadir, GH burden, T3, T4, TSH, PTH, ACTH, TC, TG, HDL, and LDL were also included in the correlation analysis and are not listed in Table 4. There were no significant correlations found between these parameters and the BMD *Z* score for any site (all *p* > 0.05).

**Figure 1:**
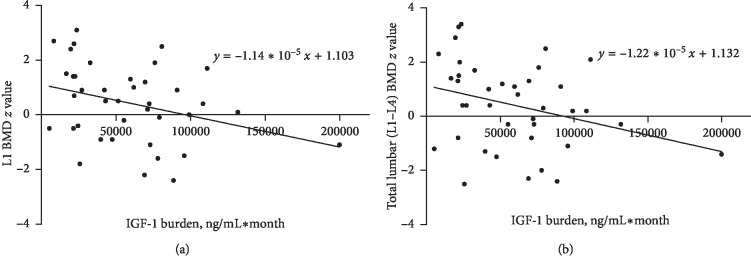
Correlation plot showing the correlations between IGF-1 burden and BMD *Z* scores: (a) a negative correlation was found between IGF-1 burden and the L1 BMD *Z* score (*r* = −0.326, *p*=0.042). (b) A negative correlation was found between IGF-1 burden and the total lumbar (L1–L4) BMD *Z* score (*r* = −0.319, *p*=0.048).

Multiple linear regression analysis showed that IGF-1 burden was negatively correlated with the preoperative BMD *Z* score at L1 (Figure 1(a)) (*r* = −0.398, *p*=0.007, *R* = 0.631) and L1–L4 (Figure 1(b)) (*r* = −0.387, *p*=0.01, *R* = 0.619) in patients with GH-secreting pituitary adenoma.

## 4. Discussion

Acromegalic osteopathy is a disease that seriously affects the quality of life of patients, and there has been no consistent conclusion regarding the effects of excess GH and IGF-1 on bone structure and bone metabolism, especially in terms of BMD changes. Our study revealed elevated BMD in patients with acromegaly, and the elevated lumbar-spine BMD recovered to a normal level after surgery. IGF-1 burden is concluded to be an independent risk factor for BMD reduction at L1. We adopted more sites for BMD measurement to obtain more comprehensive information than that yielded by the previous study. Patients with nonfunctioning pituitary adenomas were used as the control group, which could effectively reduce the interference of other potential factors.

The BMD in patients with active acromegaly was significantly elevated at L1, L2, femoral neck, Ward triangle, trochanter, femoral shaft, and total hip sites compared with that in patients with nonfunctioning pituitary adenomas. Conflicting results concerning the BMD changes in patients with acromegaly have remained over the past several decades. Previous studies by Zgliczynski et al. [5] and Kaji et al. [6] showed that BMD in the middle tibia, lumbar spine, femoral neck, and trochanter were significantly elevated in patients with acromegaly. Tuzcu et al. [7] reported that the BMD values in the lumbar spine and femoral neck of acromegaly patients were not significantly different from those in normal controls. Nevertheless, a few researchers have reported decreased BMD in patients with acromegaly [8]. On the one hand, patients with acromegaly are more likely to exhibit osteoarthritis in the spine and hip, resulting in structural changes. These changes may affect the measurement of BMD in the spine and hip [17]. On the other hand, the increase in bone area in patients with acromegaly may also lead to a decreased BMD, especially when using the DXA method [18]. The results of this study are consistent with those of most previous studies. We found that BMD in patients with acromegaly was elevated, and this change was likely to be the result of long-term chronic effects of high levels of GH and IGF-1. In previous investigations, the lumbar spine was regarded as a whole site, and the average BMD was considered. In our study, the BMD of the four lumbar vertebrae from L1 to L4 were evaluated in detail. In addition, the BMD at L1 and L2 in patients with acromegaly was elevated significantly. By comparing the *Z* score, we were more convinced that the BMD at L1 was significantly elevated, while the BMD values at L3 and L4 were not changed.

Our study also found that after 1 year of surgery, the BMD *Z* scores of the lumbar vertebrae in patients with acromegaly were significantly lower than the preoperative values, and there was no significant difference compared with the control group. It can be considered that the lumbar-spine BMD recovers to a normal level after surgery. However, the BMD *Z* score at the femoral neck continued to increase. Tamada et al. [19] showed that there was no significant difference in BMD between the timepoint of 3 years after surgery and the preoperative period in patients with acromegaly. Mazziotti et al. [20] showed that there was no significant difference in lumbar-spine BMD between the timepoint of 3 years after surgery and the preoperative period, while the femoral neck BMD was significantly reduced. In both of the above studies, hormone replacement therapy was used in some patients to treat hypopituitarism before or after TSS, while GH replacement therapy may have promoted bone formation and increased BMD [21–23], which could have covered up the BMD decrease trend. In our study, there were no patients who underwent hormone replacement therapy; thus, we can exclude the effect of hormone replacement therapy on BMD, which can reflect the changes in postoperative BMD more accurately. Since we do not have data from longer follow-up, the BMD changes over the subsequent few years are unclear. Further study will continue to focus on the postoperative BMD changes. Therefore, it can be concluded that the elevated lumbar-spine BMD in patients with acromegaly recovered to normal levels after surgery when the GH and IGF-1 levels were controlled.

In addition, the results of our study are different from those of previous studies. The postoperative BMD *Z* score decreased at the lumbar vertebrae and was elevated at the femoral neck. The difference in structure and mechanics can account for this difference. In terms of structure, the lumbar spine is rich in trabecular bone, while cortical bone is prevalent at the femoral neck [24]. BMD at trabecular bone has been reported to decrease in patients with acromegaly [8]. Other studies have shown that excessive GH and IGF-1 have a deleterious effect on trabecular bone, while GH-induced periosteal ossification increases the BMD in cortical bone [21, 25]. Therefore, we believe that the different effects of excessive GH and IGF-1 on cortical and trabecular bones result in differences in the trend of BMD between the lumbar spine and the femoral neck.

We found that the disease duration and IGF-1 burden were negatively correlated with BMD *Z* score. The multiple linear regression also showed that IGF-1 burden is an independent risk factor for reduced preoperative L1 BMD *Z* score. Previous studies have shown a negative correlation between BMD and disease duration [8], and lumbar-spine BMD is positively correlated with IGF-1 levels [7]. Some other studies have shown that lumbar-spine BMD is negatively correlated with the duration of hypogonadism, positively correlated with the duration of acromegaly, but not correlated with the IGF-1 level. Femoral neck BMD is positively correlated with the IGF-1 level and not correlated with the duration of hypogonadism and the duration of acromegaly [26]. By analysing previous studies, we believe that the increase in BMD may be due to the long-term changes in bone structure caused by excess GH and IGF-1. Our study showed that IGF-1 burden (IGF-1 level × disease duration) was negatively correlated with BMD *Z* score at the lumbar spine. Therefore, we concluded that IGF-1 burden was an excellent indicator of BMD. However, we only came to this conclusion for the BMD *Z* score at L1, and further research is waiting to be verified by using more samples and by adopting additional measurement sites.

The serum phosphorus level was significantly increased in patients with acromegaly in our study. Hyperphosphatemia is frequently observed in patients with acromegaly [27–29]. The elevated phosphorus level was slightly higher than the upper normal range. The correlation analysis showed that phosphorus level was not significantly correlated with preoperative BMD in patients with GH-secreting pituitary adenoma. Therefore, the effect of a higher phosphorus level on elevated BMD in patients with GH-secreting pituitary adenoma may not be dominant in our study. In addition, the FT3 and FT4 levels were significantly higher in patients with acromegaly than in patients with nonfunctioning pituitary adenomas in our study. Excessive GH has been reported to have effects on thyroid function, resulting in changes in thyroid hormone [30]. Pearson's correlation analysis showed that the thyroid hormone levels were not significantly correlated with BMD in patients with GH-secreting pituitary adenoma. The elevated FT3 and FT4 levels were still within the upper normal range. The T3 and T4 levels were also within normal range. A previous study on postmenopausal women showed that elevated thyroid hormones levels within the upper normal range were associated with decreased BMD [31]. Therefore, we believe that the variation in thyroid hormones levels contributed little to the increase in the BMD of patients with GH-secreting pituitary adenoma.

Our investigation has its limitations. First, this is a retrospective study, and the randomness was not fully satisfied. Therefore, the conclusions drawn from the study population did not fully represent the overall situation of patients with pituitary GH adenomas. Second is the limitation of the DXA method for BMD measurement. This method cannot well distinguish cortical bone and trabecular bone. As mentioned above, there are differences in the effects of excessive GH and IGF-1 on cortical bone and trabecular bone. Therefore, the results did not fully reflect the detailed changes in BMD. In addition, the DXA method cannot detect changes in bone microarchitecture and is easily interfered with by factors such as periosteal ossification and osteoarthritis. In contrast, qCT can measure BMD in three dimensions and can also distinguish between cortical bone and trabecular bones [32]. However, considering radiation exposure and medical expenses, the DXA method is still a good tool for observing the general trend of BMD change. Finally, the size of the study population was very limited. Due to medical expenses, traffic problems, and concerns about radiation exposure, many patients did not have regular follow-up and BMD re-examinations.

Despite the above limitations, our study can help us better understand the pathophysiological influence of excessive GH and IGF-1 on bone metabolism. As the disease duration and IGF-1 burden were negatively correlated with the preoperative BMD in patients with GH-secreting pituitary adenoma, clinical intervention on BMD should be conducted in the acromegalic patients who have higher levels of IGF-1 or longer disease duration. It is hypothesized that the BMD may be a biomarker of disease activity in patients with acromegalic osteopathy, but further studies are needed to confirm this hypothesis. Furthermore, BMD may be considered a routine examination in acromegaly patients.

## 5. Conclusions

In conclusion, the preoperative BMD in patients with acromegaly is significantly elevated at L1, L2, femoral neck, Ward triangle, trochanter, femoral shaft, and total hip sites. The lumbar-spine BMD recovered to normal levels after successful surgery when GH and IGF-1 levels were controlled. Preoperative lumbar-spine BMD *Z* score was negatively correlated with disease duration and IGF-1 burden, and IGF-1 burden was an independent risk factor for preoperative BMD *Z* score decrease at L1 in patients with acromegaly.

## Figures and Tables

**Table 1:** Basic information of the GH-secreting pituitary adenoma group and the nonfunctioning pituitary adenoma group.

	GH-secreting pituitary adenoma	Nonfunctioning pituitary adenoma	*p*
*N*	39	29	—
Gender (M/F)	19/20	15/14	—
Age (years)	43.74 ± 10.33	49.14 ± 16.00	0.120
BMI (kg/m^2^)	26.03 ± 4.57	24.69 ± 2.73	0.292
Disease duration (months)	72.15 ± 49.33	17.97 ± 34.84	<0.001
Adenoma size (mm)	15.97 ± 6.87	26.43 ± 9.63	<0.001
GH (ng/mL)	33.49 ± 48.75	0.80 ± 1.77	<0.001
IGF-1 (ng/mL)	855.00 ± 261.91	168.52 ± 81.14	<0.001
T3 (ng/mL)	1.37 ± 1.47	0.97 ± 0.22	0.109
T4 (ng/mL)	7.64 ± 1.70	7.45 ± 1.64	0.660
FT3 (pg/mL)	3.25 ± 0.70	2.65 ± 0.45	<0.001
FT4 (pg/mL)	1.17 ± 0.21	1.06 ± 0.20	0.041
TSH (mU/L)	1.55 ± 1.03	2.33 ± 2.59	0.143
F (ng/mL)	10.12 ± 5.22	12.43 ± 6.29	0.130
ACTH (pg/mL)	38.12 ± 23.96	26.39 ± 18.20	0.095
PRL (ng/mL)	21.34 ± 40.13	23.30 ± 20.03	0.796
T (ng/mL)	1.41 ± 1.07	1.75 ± 1.62	0.740
P (ng/mL)	1.30 ± 2.78	0.59 ± 0.41	0.237
E2 (pg/mL)	60.89 ± 121.74	103.63 ± 263.72	0.497
LH (IU/L)	10.82 ± 10.74	8.66 ± 8.04	0.303
FSH (IU/L)	22.77 ± 24.69	21.52 ± 22.67	0.860
Ca (mmol/L)	2.36 ± 0.18	2.33 ± 0.10	0.403
P (mmol/L)	1.55 ± 0.22	1.26 ± 0.13	<0.001

BMI: body mass index; GH: growth hormone; IGF-1: insulin-like growth factor-1; T3: triiodothyronine; T4: thyroxine; TSH: thyroid-stimulating hormone; F: cortisol; ACTH: adrenocorticotropic hormone; PRL: prolactin; T: testosterone; P: progestogen; E2: oestradiol; LH: luteinizing hormone; FSH: follicle-stimulating hormone; Ca: calcium; P: phosphorus.

**Table 2:** BMD of the GH-secreting pituitary adenoma group and the nonfunctioning adenoma group.

	GH-secreting pituitary adenoma	Nonfunctioning pituitary adenoma	*p*
L1	1.107 ± 0.187	0.998 ± 0.124	0.006
L1-Z	0.433 ± 1.414	−0.186 ± 1.116	0.048
L2	1.159 ± 0.213	1.067 ± 0.132	0.032
L2-Z	0.310 ± 1.654	−0.190 ± 1.110	0.141
L3	1.228 ± 0.220	1.151 ± 0.134	0.078
L3-Z	0.562 ± 1.630	0.279 ± 1.234	0.419
L4	1.200 ± 0.227	1.149 ± 0.155	0.271
L4-Z	0.344 ± 1.720	0.231 ± 1.392	0.767
L1–L4	1.178 ± 0.208	1.097 ± 0.125	0.053
L1–L4-Z	0.418 ± 1.583	0.059 ± 1.150	0.282
L2–L4	1.197 ± 0.216	1.124 ± 0.131	0.092
L2–L4-Z	0.408 ± 1.642	0.128 ± 1.202	0.420
Femoral neck	1.034 ± 0.161	0.906 ± 0.133	0.001
Femoral neck-Z	0.882 ± 1.120	0.332 ± 0.796	0.022
Ward triangle	0.815 ± 0.165	0.723 ± 0.179	0.036
Ward triangle-Z	−0.023 ± 1.047	−0.232 ± 0.830	0.366
Trochanter	0.834 ± 0.141	0.746 ± 0.111	0.005
Trochanter-Z	0.346 ± 1.129	−0.082 ± 0.855	0.082
Femoral shaft	1.207 ± 0.206	1.118 ± 0.136	0.037
Total femora	1.034 ± 0.164	0.940 ± 0.119	0.008
Total femora-Z	0.531 ± 1.196	0.193 ± 0.787	0.168

**Table 3:** Postoperative BMD *Z* score changes in patients with acromegaly.

	Before surgery	After surgery	Control	P1	P2
L1-Z	0.600 ± 1.134	0.231 ± 1.176	−0.186 ± 1.116	0.037	0.292
L2-Z	0.546 ± 1.385	−0.046 ± 1.212	−0.190 ± 1.110	0.006	0.720
L3-Z	0.500 ± 1.466	0.115 ± 1.289	0.279 ± 1.234	0.029	0.703
L4-Z	0.477 ± 1.705	0.008 ± 1.552	0.231 ± 1.392	0.031	0.661
L1–L4-Z	0.539 ± 1.433	0.046 ± 1.279	0.059 ± 1.150	0.012	0.976
L2–L4-Z	0.508 ± 1.532	0.023 ± 1.357	0.128 ± 1.202	0.012	0.814
Femoral neck-Z	1.023 ± 1.073	1.269 ± 1.085	0.332 ± 0.796	0.019	0.012
Ward triangle-Z	0.231 ± 1.154	0.331 ± 1.261	−0.232 ± 0.830	0.240	0.160
Trochanter-Z	0.415 ± 1.049	0.477 ± 1.042	−0.082 ± 0.855	0.619	0.107
Total hip-Z	0.585 ± 1.130	0.777 ± 1.173	0.193 ± 0.787	0.040	0.121

The patients with nonfunctioning pituitary adenoma were regarded as the control. P1: *p* value of the comparison between preoperative and postoperative BMD; P2: *p* value of the comparison between the postoperative and control group.

**Table 4:** Correlation analysis of clinical data and the BMD *Z* score.

		L1	L2	L3	L4	L1–L4	L2–L4	Femoral neck	Ward triangle	Trochanter	Total hip
Age (years)	*r*	−0.110	−0.125	−0.156	−0.125	−0.123	−0.131	−0.017	0.068	0.056	−0.039
	*p*	0.506	0.449	0.343	0.447	0.456	0.425	0.919	0.682	0.735	0.816
BMI (kg/m^2^)	*r*	−0.049	−0.048	0.069	−0.034	−0.014	−0.023	0.082	−0.102	0.054	0.235
	*p*	0.768	0.772	0.675	0.835	0.931	0.887	0.618	0.535	0.742	0.150
Disease duration (months)	*R*	−0.389^*∗*^	−0.370^*∗*^	−0.371^*∗*^	−0.371^*∗*^	−0.413^*∗*^	−0.393^*∗*^	−0.280	−0.276	−0.319^*∗*^	−0.324^*∗*^
	*p*	0.014	0.021	0.020	0.020	0.009	0.013	0.084	0.088	0.048	0.044
Adenoma size (mm)	*r*	0.070	0.183	0.144	0.147	0.139	0.155	0.102	0.120	0.104	0.114
	*p*	0.670	0.264	0.383	0.371	0.400	0.346	0.535	0.466	0.528	0.488
GH (ng/mL)	*r*	0.240	0.272	0.186	0.178	0.229	0.214	0.194	0.101	0.191	0.192
	*p*	0.140	0.094	0.257	0.277	0.161	0.190	0.236	0.541	0.245	0.241
IGF-1 (ng/mL)	*r*	0.021	0.097	0.097	0.096	0.086	0.106	0.191	0.041	0.133	0.193
	*p*	0.897	0.556	0.559	0.563	0.603	0.522	0.244	0.802	0.421	0.240
IGF-1 burden (ng/mL *∗* month)	*r*	−0.326^*∗*^	−0.273	−0.277	−0.267	−0.319^*∗*^	−0.298	−0.150	−0.197	−0.213	−0.174
	*p*	0.042	0.093	0.088	0.100	0.048	0.066	0.362	0.230	0.194	0.291
FT3 (pg/mL)	*r*	−0.006	−0.077	−0.066	−0.057	−0.072	−0.073	−0.215	−0.231	−0.128	−0.117
	*p*	0.970	0.643	0.692	0.729	0.665	0.659	0.188	0.157	0.436	0.477
FT4 (pg/mL)	*r*	−0.195	−0.176	−0.081	−0.096	−0.124	−0.118	−0.100	−0.057	−0.095	−0.107
	*p*	0.234	0.283	0.623	0.559	0.452	0.474	0.543	0.728	0.564	0.518
PRL (ng/mL)	*r*	−0.129	−0.184	−0.219	−0.240	−0.227	−0.234	−0.329^*∗*^	−0.255	−0.361^*∗*^	−0.313
	*p*	0.435	0.263	0.180	0.142	0.164	0.151	0.041	0.117	0.024	0.053
Ca (mmol/L)	*r*	0.140	0.057	0.038	0.001	0.083	0.054	0.230	0.250	0.233	0.136
	*p*	0.396	0.730	0.819	0.995	0.614	0.746	0.159	0.124	0.154	0.411
P (mmol/L)	*r*	0.077	0.048	0.040	−0.039	0.024	0.015	−0.015	−0.022	−0.025	0.042
	*p*	0.641	0.771	0.808	0.812	0.883	0.929	0.929	0.897	0.878	0.801

BMI: body mass index; GH: growth hormone; IGF-1: insulin-like growth factor-1; FT3: free triiodothyronine; FT4: free thyroxine; PRL: prolactin; Ca: calcium; P: phosphorus.

## Data Availability

All data included in this study are available upon request by contact with the corresponding author.
